# Challenges and opportunities for breast cancer early detection among rural dwelling women in Segamat District, Malaysia: A qualitative study

**DOI:** 10.1371/journal.pone.0267308

**Published:** 2022-05-20

**Authors:** Désirée Schliemann, Wilfred Mok Kok Hoe, Devi Mohan, Pascale Allotey, Daniel D. Reidpath, Min Min Tan, Nur Aishah Mohd Taib, Michael Donnelly, Tin Tin Su

**Affiliations:** 1 Centre for Public Health and UKCRC Centre of Excellence for Public Health, Queen’s University Belfast, Belfast, United Kingdom; 2 South East Asia Community Observatory (SEACO), Jeffrey Cheah School of Medicine and Health Sciences, Monash University Malaysia, Segamat, Malaysia; 3 Global Public Health, Jeffrey Cheah School of Medicine and Health Sciences, Monash University Malaysia, Bandar Sunway, Malaysia; 4 International Institute for Global Health, United Nations University, Kuala Lumpur, Malaysia; 5 International Centre for Diarrhoeal Disease Research, Dhaka, Bangladesh; 6 Faculty of Medicine, Department of Surgery, Universiti Malaya Cancer Research Institute, University of Malaya, Kuala Lumpur, Malaysia; Universiti Teknologi Malaysia, MALAYSIA

## Abstract

**Introduction:**

Breast cancer patients in low- and middle-income countries often present at an advanced stage. This qualitative study elicited views regarding the challenges and opportunities for breast cancer screening and early detection among women in a low-income semi-rural community in Segamat district, Malaysia.

**Methods:**

Individual semi-structured interviews with 22 people (health professionals, cancer survivors, community volunteers and member from a non-governmental organization) and four focus group discussions (n = 22 participants) with women from a local community were conducted. All participants were purposively sampled and female residents registered with the South East Asia Community Observatory aged ≥40 years were eligible to participate in the focus group discussions. Data were transcribed verbatim and analyzed using thematic analysis.

**Results:**

The thematic analysis illuminated barriers, challenges and opportunities across six domains: (i) personal experiences and barriers to help-seeking as well as financial and travel access barriers; (ii) primary care challenges (related to delivering clinical breast examination and teaching breast-self-examination); (iii) secondary care challenges (related to mammogram services); (iv) disconnection between secondary and primary care breast cancer screening pathways; and (v) opportunities to improve breast cancer early detection relating to community civil service society activities (i.e. awareness raising, support groups, addressing stigma/embarrassment and encouraging husbands to support women) and vi) links between public healthcare personnel and community (i.e. improving breast self-examination education, clinical breast examination provision and subsidised mammograms).

**Conclusion:**

The results point to a variety of reasons for low uptake and, therefore, to the complex nature of improving breast cancer screening and early detection. There is a need to adopt a systems approach to address this complexity and to take account of the socio-cultural context of communities in order, in turn, to strengthen cancer control policy and practices in Malaysia.

## Introduction

Breast Cancer (BC) is the most common cancer amongst females in Malaysia with an age-standardised incidence rate (ASR) of 34.1/100,000 [1]. Late-stage presentation is a major challenge in Malaysia and has increased from 43.2% between 2007–2011 to 47.9% between 2012–2016 [1]. BC screening of asymptomatic women is key to detecting and treating cancer early and improving outcomes for cancer patients. Mammography and clinical breast examination (CBE) are the most common BC screening methods globally. Mammography is an x-ray imaging method used to examine breasts for tumours. BC screening is opportunistic in Malaysia and the Ministry of Health recommends for women in the general population aged 50–74 years to be offered mammography biannually [2]. CBE is a physical exam of the breast by clinical staff to check for lumps or other signs of BC. It is a low-cost screening method that, if performed well, achieves the same effect as mammography in terms of mortality [3]. Opportunistic screening requires health care professionals to prompt women who attend the clinic for other reasons to be screened for BC or for women to notice a change in their breast and seek help. Breast self-examination (BSE) is commonly taught to women to regularly self-check for abnormal signs and symptoms, in particular lumps.

Despite these recommendations and efforts to educate women about BC and BC screening, mammography screening in Malaysia has been a particular challenge in rural communities and remains low despite efforts to implement mobile screening camps [4,5], i.e. it has been reported to range from 6.8% to 8.3% in rural areas and from 8.3% and 15% in urban and sub-urban areas [6]. Disparities in BC screening between women from rural/urban areas as well as from different socio-economic backgrounds have been observed around the world but with greater differences in low- and middle- income countries (LMICs) [7–10]. An in-depth understanding of the unique challenges that women as well as health care providers from rural areas face in providing BC screening is necessary to address the disparities in BC screening uptake in Malaysia. Previous studies that investigated barriers to BC screening uptake have mainly been of quantitative nature [11–14] and some qualitative studies have explored perceived barriers to screening in breast cancer patients [15]. It has been suggested that stigma associated with BC, fear of diagnosis, lack of knowledge, financial concerns and cultural concerns are common barriers to screening [6,16]. Views of women with no BC history within semi-rural communities have not been explored qualitatively. Similarly, there is a lack of understanding of health system challenges and opportunities perceived by health professionals in Malaysia. The scope of this research included several studies about BC screening and its uptake in Segamat District of Malaysia. The results presented in this paper focus on the qualitative exploration of the challenges, barriers and opportunities that women and health care professionals describe regarding BC screening in Malaysia.

## Methods

This was a qualitative study based on focus group discussions (FGDs) with women from semi-rural communities in Malaysia and semi-structured interviews with key stakeholders that were conducted between July and September 2017. Purposive sampling was used to select participants. All participants provided written informed consent prior to each interview/FGD. We followed the consolidated criteria for reporting qualitative research guidelines (COREQ) to report the conduct and results of this study [17].

### Setting

Malaysia is an upper middle-income country located in South-East Asia. Its multi-ethnic population of 32.4 million people is made up of 70% Malays, 23% Chinese, 7% Indian, and 1% of other ethnicities [18]. This study was conducted at the South East Asia Community Observatory (SEACO), a health and demographic surveillance system located in Segamat district, in the state of Johor in Peninsular Malaysia [19]. SEACO operates in 5 of the 11 sub-districts in Segamat, covering an area of approximately 1250 km^2^. The ethnic representation of the SEACO population is fairly representative, i.e. 62% Malay, 18% Chinese, 10% Indians, 2% indigenous peoples (Orang Asli), and others (8%) [19]. Participants for this study were selected from semi-rural settings under SEACO and recruited over the phone.

### Focus group discussions

The objectives of the FGDs were to explore the community’s understanding and perceptions of BC, BSE, CBE and mammogram screening, as well as perceived challenges to attend BC screening. Women aged ≥40 years from SEACO residents were eligible to participate. Participants were identified through the SEACO community engagement committee [20]. A total of 22 multi-ethnic women participated in four face-to-face, semi-structured FGDs that were held in local community centres. A trained female anthropology student conducted the FGDs together with a trained female SEACO research staff who spoke Malay and Mandarin. Hand-written verbatim notes were produced in addition to the audio recordings. The sessions were recorded, transcribed and translated into English. Each FGD began with an initial round of introductions, an explanation of the topic and research objectives, followed by guided FGDs that lasted up to one hour.

### Individual semi-structured interviews

The objectives of these interviews were to explore perceptions about BC services, utilization levels of the services and perceived challenges faced in delivering the services. The interview topic guides were developed based on expert panel discussions and a literature review. Twenty-two people participated in semi-structured interviews, including staff nurses under the Ministry of Women, Family and Community Development (LPPKN), physicians from the breast clinic, nurses, a radiologist and a radiographer from a secondary district hospital, doctors and nurses from community and district health centres, general practitioners (from private clinics), volunteers from the Breast Cancer Support Society Segamat (BCSS), community volunteers and BC survivors. A trained female anthropology student conducted all interviews in English and a female SEACO research staff produced hand-written verbatim notes in addition to the audio recordings. Interviews were conducted in participant homes/offices and lasted approximately 40 minutes. All interviews were audio-recorded and transcribed.

### Data analysis

A thematic analysis was conducted in NVivo vs 12. Three authors (WMKH, DS and MMT) coded the interviews independently, compared the codes and identified sub- and key-themes. Any disagreements were discussed with co-authors until an agreement was reached.

### Ethical approval and consent

This study received ethical approval from the Monash University Human Research Ethics Committee (ID 29682) and Malaysian Medical Research and Ethics Committee (NMRR-17-1244-35902). Consent to participate was gained verbally during the telephone recruitment interview.

## Results

The separate analysis of the individual interviews and FGDs generated the same themes and, so, they are presented together. Table 1 shows the background of participants who completed the interview and Table 2 demonstrates the socio-demographic background of FGD participants. The data analysis generated six key themes that revolved around challenges (n = 3), barriers (n = 1) and opportunities (n = 2).

- Primary care limitations (in terms of the extent to which it can provide gender sensitive and targeted CBE screening services)
- Challenges in providing mammograms in public sector secondary care hospitals
- A disconnection between primary care-oriented BC screening pathway and secondary care)
- Personal experiences and barriers to help-seeking
- Community and civil society activities
- Links and networks between public healthcare personnel and community.

**Table 1:** Participation in individual interviews.

Interviews	Participants (n)
Breast cancer survivors	4
Community volunteers	4
General practitioners	3
District hospital (doctors, radiographer, nurses)	4
District health clinics (doctors & nurses)	3
Breast cancer support society (BCSS) members	2
Staff nurses from the National Population and Family Development Board Malaysia under the Ministry of Women, Family and Community Development (LPPKN)	2
**Total**	22

**Table 2:** Socio-demographic information from FGD participants (n = 22).

	n	%
**Age**		
30–39	1	4.5
40–49	9	40.9
50–59	7	31.8
60–69	4	18.2
≥70	1	4.5
**Ethnicity**		
Malay	5	22.7
Chinese	5	22.7
Indian	6	27.3
*Orang Asli*	6	27.3
**Education** ^**a**^		
No formal education	3	13.6
Primary	8	36.4
Secondary	7	31.8
Tertiary	1	4.5
Missing	3	13.6
**Household income**		
< RM 1,000	8	36.4
RM 1,000 –RM 4,000	11	50.0
Missing	3	13.6
**Sub-district**		
Sungai Segamat	4	18.2
Gemereh	5	22.7
Bekok	6	27.3
Chaah	6	27.3
Pogho	1	4.5
**Distance to nearest clinic (km)**		
<5km	16	72.7
5-10km	0	0
≥10km	6	27.3

Each theme is discussed here and relevant quotes are presented in Table 3.

**Table 3:** Key- and sub-themes identified in the focus group discussions and interviews.

Key themes	Sub-themes	Quotes
1. Challenges		
1.1 Primary care limitations (in terms of the extent to which it can provide gender sensitive and targeted CBE screening services)	Insufficient staff resources and high workload	*I do it myself to an extent I wanted to cry (laugh)*, *so that many people would come here*. *Last time I only had 2 staffs*, *with my assistant*, *I do everything alone*. *Last year is very*, *my bad year (laugh)*. *Even for school holiday*, *I didn’t take any leave*, *my children is off school for one month*, *just sit here with me (laugh)*, *from morning until*, *uh*, *until end of the month when we have written mail*, *I struggled*, *even one day*, *full week*, *I had to work from 8 AM in the morning until 8PM*! *PM*! *I stayed here from 8AM until 8PM*! *Last time*, *until I cried (laugh)*.–LPPKN nurse
	Long waiting times	*‘Older people*. *They prefer home visits*. *In the clinic or the hospital*, *the wait is long*, *they don’t want to waste their time*.*’*–FGD Malay
	Unavailability of female staff for CBE	*‘… Malay people*, *if they clinic only has male doctors*, *they will feel embarrassed’*–FGD Malay *‘…There are a lot of male doctors*. *The women are afraid to expose their bodies*.*’*–FGD Indian *‘… unfortunately we don’t have enough female doctors*, *I think ideally it should be helped by more female doctors*, *I don’t many female early in age group or even in the middle age group would like to volunteer themselves to go for medical examination*. *Of course we can teach them*, *but you can teach them*, *ideally again it would be a women*, *a women doctor or at least a nurse*, *a nurse background*, *so we don’t have that structure’*–Private clinic doctor
	Variable CBE clinical skills	*‘Yes*, *the nurses are trained for the breast examination*, *but mainly to educate*, *they were trained on how to educate the people*, *but whether they can pick up the lump or not*, *the senior one definitely*, *the junior ones are still lacking in experience*.*’*–Government clinic doctor
	CBE in clinics for at-risk women not the norm (often when patient symptomatic or opportunistic in antenatal clinics)	*‘…we only check when they complain about it*! *If they didn’t we wouldn’t know*! *If there’s a wound we can see*. *They complain*, *I am not feeling well*, *only then we can check*.*’*–Government clinic doctor *‘Anytime patient can walk in for the clinical breast examination*, *mainly it’s just that when patients have their babies*, *they come for antenatal check-up*, *then we will educate them on breast self-examination*. *Then the patient will do that at home*, *and they have problem they come in*, *there’s no special dates or special clinics for that*. *But this is outdated way*, *some of the recommendation*, *is uh*, *actually don’t recommend that anymore*.*’*–Government clinic doctor
	BSE taught mainly to women of child-bearing age	*‘…any patient coming to mother and child*, *they teach breast examination’*–Government clinic doctor
	Inadequate teaching about BC and BSE during community screening programmes due to societal norms (no physical contact)	*‘We are a conservative country*, *we don’t normally demonstrate it for [the women]*,* normally they just show it through pictures*, *especially during health screening programmes*. *The most that we do is just show them the breast which have lesions*, *so let them try to have that touch*, *that feeling*, *what kind of lesion it is*, *that is the maximum that we did*, *but show them exactly how to do hands-on*, *no*.*’* Government clinic doctor *I think maybe they have less exposure like maybe to the social media*, *because usually it’s the poster that say you need to examine like this and like that*, *but they don’t have a real person to teach them*, *the hands-on*, *how they going to demonstrate*.–Private clinic doctor
	Lack of suitable space for community outreach	*‘But this is a problem because usually these things are in an open space*, *there is no privacy to do BSE*. *These things are done by paramedics*.*’*–Government clinic doctor
1.2 Challenges in providing mammograms in public sector secondary care hospitals	Unavailability of (female) staff specialised in breast cancer care	*‘We have 14*, *only 4 of us are female radiographer*, *it also involves shift timetable*, *so sometimes female radiographer works at night*, *she will be working in the afternoon*, *so we lack of staff*.’–Radiographer ‘*During that time*, *when we have palliative*, *we don’t have staff*, *staff who are at hospital are member of this palliative team*. *So*, *indeed we are short of staff*. *Sometimes it is related to doctor*, *if we don’t have doctor*, *then there’s no staff*.’–Government hospital doctor *‘… the country is still lacking specialists*, *[…] the pool of medical officers will be getting more and more […]*. *Say you have 5 hospitals and you have 5000 doctors*, *okay*, *but talking about all the other supportive staffing things like nurses*, *it’s actually still lacking*.*’*–Government hospital doctor
	Lack of funding to subsidise mammograms	*“They have subsidy for doing mammogram at private*, *however the subsidy takes time too*, *for their appointment*. *Not everyone can get it*. *They have their quota*, *only up to a number of patients can be sent for mammogram only*.”–Government hospital nurse *However*, *the situation is different too compared to the past*, *we didn’t have limited quota*. *Right*. *Many*, *1000*, *sometimes it reaches 300 of our*, *what’s that*, *the registration*. *However*, *this year*, *we followed the budget from 2017*, *they only allocate*, *1Malaysia clinic*, *how many of them*? *15k or how many*?—LPPKN nurse *Because of budget from the government*. *Prime Minister’s budget*. *Cutting budget*. *For last time*, *unlimited budget*, *so*, *quota*, *we don’t have quota*.—LPPKN nurse
	Poor maintenance of facilities (not just the equipment)	*‘…the mammogram at Muar*, *if the air-con has problems you cannot run the machine right*? *Doesn’t mean machine cannot be switched on*, *machine is okay*, *but the air-con is problematic*, *so you can’t turn the machine*. *It happened before*. *Furthermore*, *it’s an old machine*. *That’s the issue*.*’*—Government hospital doctor
	Poor patient information about mammogram screening	‘*If you to send out people to Pantai*, *they just do the mammogram and give them the slide and that’s it*. *They don’t explain to them*. *But if you send them to IIUM*, *they will explain*, *on the screen*, *on the*, *the … what you call*? *The x-ray*.’–BCSS member Interviewer: *During your mammogram visit […]*, *do the doctors and nurses provide deeper explanations*? FGD (Indian): *We cannot take so much information*. Interviewer: *Have you ever gone for a mammogram*? FGD (Indian): *Once*, *but we didn’t know what it was for*.
1.3 A disconnection between primary care-oriented BC screening pathway and secondary care	Competing government-set KPIs in different departments (inform budget for funded mammograms/ CBEs and motivate clinics to offer opportunistic screening)	*‘In fact initially also you will have a bit of hiccup [with providing clinical breast examination] because you have a KPI in the government health clinic*, *I only knew about this after we started running this centre*, *so they have a KPI of how many breast lumps that your clinics must examine*, *you know*? *That’s why I said*, *a lot of times*, *this come with policy maker*, *so as a policy maker*, *so you really need to understand what the groundworks are running*.*’*–Government hospital doctor *‘…you wait for them to realise there is a breast lump*, *then they will normally go and seek for consultation from the GPs*, *and GP will write a referral letter for them to see a surgeon in the hospital*, *will be referred to hospital*. *If you have been referred to a public hospital*, *most of the time under the surgical arm there will be a breast clinic*, *there will be a breast surgery clinic*, *or even if they don’t have a breast surgery clinic*, *they will see you under general surgery clinic*, *so most of the time from the previous year our experience is uh*, *when the patient went to the hospital with the referral letter*, *they will be given an appointment*, *which according to the KPI will be roughly about uh*, *not more than 1 month*, *so they will be given an appointment not more than 1 month*, *then after that you see the doctor*, *then the doctor feel that it’s a necessary for you to go through certain imaging*, *then they will book you up another appointment’—*Government hospital doctor *‘In Malaysia*, *you have a lot of all these policy*, *so there you have a little bit of hiccup*, *people thought you want to take over their job totally*, *right*? *So how about my KPI*? *My KPI would drop […] if you bypassed us to refer yourself to the BCare*, *but for us*, *standing on the other end of treatment*, *the ultimate people who are going to do to the surgery and going to treat the patient*, *we think that a lot of time all this initial seeing is unnecessary*, *we want to get to the patient as soon as possible to initiate the treatment*, *because we know very well that early detection and early treatment give you a better outcome*! *So that also having some hiccup*, *but I think after some years*, *they also understand very well*.*’*–Government hospital doctor
**2. Barriers**		
Personal experiences and barriers to help-seeking	Poor awareness and understanding about BC screening	*‘Because*, *another thing is that many doesn’t understand what mammogram is*, *they don’t understand and don’t want to know about it*, *the people from kampong right*, *women*, *because this is for those above 40 years old*, *like those elderly women with grandchildren*, *aged more than 40*, *so they tend not to want it*, *because they didn’t study much*, *so they’re like “what is that thing*, *don’t need it*, *don’t want*!’–LPPKN nurse *‘Some said if they did this x-ray*, *it will kill the cells*, *so they are afraid of getting this*, *they are like negative*. *Difficult too*.*’*–LPPKN nurse *‘I am very sad is the*, *many people still don’t know about breast cancer*.*’*–Cancer Survivor
	Low perceived susceptibility	*‘…but they feel that they won’t have it*, *won’t have breast cancer*, *so they don’t have to check or do anything*.*’*–Government clinic doctor
	Preference for traditional treatment due to fear or lack of knowledge about medical procedures	*Sometimes*, *they are afraid of the diagnosis*. *They would rather resort to a traditional healer*.—FGD Malay *Yes*. *Most of my patients there don’t have legal documentation*, *they are Malaysians but they don’t have that*, *so they don’t have all these knowledge to go and see the doctor*. *They will go and see the bomoh (Shaman) first*, *then they will treat with all funny things*, *and then they come to us*, *that’s in late stage already*.–Private clinic doctor *We only visit the traditional doctor when modern medicine isn’t effective*.—FGD Malay *‘…majority of Orang Asli will use traditional medication*, *whether it’s bomoh (Shaman) or herbs*. *So indeed these are passed down for generations*, *until now*, *I’m not sure about other villages*, *but my village*, *there are some who still practice taking traditional medication*.*’*–Government clinic doctor
	Financial concerns	*Last time we used to go to Kuantan*, *so they give us subsidies to come from KL*, *so we have a van*, *we can take up 30 people*, *serve the public*, *and then they just pay RM30 for mammogram*, *and we all cancer patient free (laugh)*, *transportation RM30*, *because we have a van*, *we have to get a driver*, *but now*, *we heard that we have a mammogram there*, *all refuse to go*, *because now they are not going to subsidise*, *because last time starting they give us 500 people every year*, *after that they reduce to 300*, *last year*?—Cancer survivor
	Access barriers (transportation; multiple visits)	*Because the patient needs to come to us first*, *and fill up the form*, *upon completion they have to set the date to go*. *Sometimes*, *for those who drive they won’t mind*, *they choose the date*, *those who put on hold*, *ask husband or children first*, *to bring them there*, *so we had to put in the mammogram form first*, *and wait for them to call us back to confirm the date*, *only then we call the centre*, *it’s a bit troublesome*. *Quite difficult*!—LPPKN nurse *‘…not everyone will come*, *some are easy to come by the clinic and collect medicine*, *the other half is rather difficult for them to visit the clinic*.*’*–Government clinic doctor *‘Because some of them*, *not most of them*, *have their own transport*. *Some of them have to rent a taxi*, *RM50 per travel*, *RM100 just for them to come to clinic*.’–Government hospital doctor *“Sometimes they cannot come because of the vehicle*, *no transport*. *Sometimes I go and fetch them*. *They are poor and have a transportation problem*.*”*–Community Volunteer
	Ethnic differences (awareness, interest in screening, preference for doctor, willingness to pay)	*For Malay people*, *if we need to be examined*, *we must see a female doctor*. *Not like Chinese or Indian people–they can see anyone*. *If we go*, *and there is only a male doctor*, *we will go home*. *They don’t allow us*.—FGD Malay *Yes*, *I think we face the same issues*. *But for breast cancer*, *I think Chinese and Indian people are more open minded*.—FGD Malay *Usually there are more Chinese*, *awareness are higher among them*. *They are quicker*, *when there is something free*, *usually they are the one who act fast (laugh)*, *compared to Indian*.—LPPKN nurse *Economic differences uh*, *how to say*, *if Chinese*, *even though they are not so well-being*, *they will still go for private because they want to fastest treatment*, *and the if Malays and Indians*, *it depends*, *some they chose for government some chose private*.–Private clinic doctor
	Language/ literacy and health literacy barriers (materials are not tailored to address them)	*I think media of course play a role*, *they go to all health clinics*, *all these brochure are available*, *uh…*.*probably it’s not available in the language they can read*.*”*–Community Volunteer *“Because the education level is also low*. *So they may find it difficult to question or follow such talks*. *So if the expert can give this in the language they understand*, *then can be easily received*.*”*–Community Volunteer
	Poor support from husband who is often the main decision maker	*“Ah*, *they don’t come…we have our organizations*, *so the earlier about the health education to the husbands… we do call the husbands*, *they say no*, *no*, *no lah*, *I’m not interested*.*”*–Community Volunteer *Embarrassed and afraid*. *They are afraid of the diagnosis*, *that they may have to remove a breast*. *Then*, *their husband will find another woman*. *But for us–we are old*, *so if we don’t have one*, *that’s alright*. *But it’s different for the younger women*.—FGD Malay *So even you put a prosthesis from outside*, *you still look fine but the issue is that they have difficulty facing their family*, *so some of my patient in Sabah*, *they said they refused to get treated because they are worried their husband are going to get a second or third wife*. *But I said if like this also I think your husband won’t want you right*? *You may die because of this*, *so they are a abit reluctant on that*.–Private clinic doctor *"Sometimes*, *the husbands do not allow us to show our bodies to another man*, *even a doctor*. *Sometimes if we mentioned we are not feeling well*, *the husband would say it is a little thing only*.*"*—FGD Malay *‘I think the Malay and Indian*, *mostly the Malay they will follow the husbands*, *sometime*, *they won’t get permission from the husband*, *the family*, *you know*.’–BCSS member
	Competing priorities (child care)	*‘Malay take care of grandchildren*. *So they have no time*. *They come out*, *sometimes they say*, *okay*, *if you do it on Friday*, *im free*. *The mother can take the baby*, *they say to me*. *So*, *sometimes we do like that*, *Friday*.*’*–Community Volunteer ‘*One or two backed out because they couldn’t get somebody to babysit their children*. *So they said they cannot*.*’*–Community Volunteer
	Embarrassment (to talk about BC and to show body to male doctor), need husband’s permission	*‘I always said that*. *For Orang Asli*. *Sometimes they felt embarrased to talk about it*.’–Government clinic doctor *‘If the clinic only has male doctors*, *we will feel embarrassed*. *It is mostly a feeling of embarrassment*.*"–FGD with the Malay community members*?*’*—FGD Malay *‘Even if a woman has breast cancer*, *sometimes they are shy to see the doctor*. *There are a lot of male doctors*. *The women are afraid to expose their bodies*.’–FGD Indian *‘Sometimes*, *the husbands do not allow us to show our bodies to another man*, *even a doctor*.*’*—FGD Malay
	Fear of health checks (i.e. fear of pain and results)	*‘But you know old people–even if there are free health checks*, *they aren’t very willing to attend*. *They’re scared*. *When they find out that they’re sick*, *they get scared*.’ FGD Malay *‘First*, *[the Orang Asli] are afraid*, *indeed*, *second*, *they are embarassed*. *They always said "I’m so scared"*, *definitely will say so*.’ –Government clinic doctor *‘…she asked me*, *is it very painful*? *I said no*, *it;s okay*, *Actually*, *it’s a little pain*, *I always tell them*, *you beat your hand is is painful*? *So*, *I said no*, *just a bit*, *doesn’t matter*.*’*—Cancer survivor *‘Yeah*, *many said they are afraid*, *afraid of pain*, *and also the results*, *fear of having the bad results*.*’*–LPPKN nurse *‘They are afraid that this machine will cause pain*.’ LPPKN nurse
	Fear of treatment (fear of losing hair and losing breast)	*‘One thing that I promote yes*, *maybe one other thing that we should promote is not only that ugly picture of breast cancer*, *we should show them the breast reconstruction also*. *Because normally the women are very pantang (forbidden) [talking] about the breast because [if] you don’t have a breast anymore then you are not a women anymore*. *So they are reluctant to get treatment’*–Private clinic doctor *‘This is important or your hair*! *There are a few you know*, *my hair dropped*, *then I don’t want*. *Eh*, *then I always have to explain to them*, *why you need chemo*. *So many people say don’t go for chemo*, *I have to explain to them*, *why you need chemo*. *And then some of them*, *I know it’s very frightening*, *I want to go to op*, *but I am sure you know mastectomy*, *how do they operate*.*’*—Cancer survivor *‘Sometimes I have to show my breast*. *Take it out*, *you see just only one mark and no stitches*, *so they said*, *there were few in the Bcare there*, *I showed them*, *oh like that only*? *Ok*, *then I don’t mind to do*.*’*—Cancer survivor
**3. Opportunities**		
3.1 Community civil society activities	Community education to raise awareness about BC and screening	*‘We do all talks and awareness campaign whereby we teach them how to do BSE*. *It is very important for finding the initial stage*, *early detection*. *In this way we would teach them the way*. *So we would be organising awareness campaigns in schools or in anywhere*.*’*–BCSS member *‘We normally do the health talks in a place*. *We ask the woman to assemble in a particular house*. *The number is not many*, *maybe five*. *They came and we gave a demonstration and teach them on breast self-examination*.*’*–Community Volunteer *‘[NGOs] can set a day which they organise the campaign at offices or medical camp at the rural area*, *to raise awareness*, *give talks*, *increase knowledge among our target groups for them to understand how to detect breast cancer earlier*. *More talks and health campaigns to let our society truly understand about BC*.*’*–District hospital nurse *‘So the breast self-examination awareness is less*, *cause there is no body to tell*, *they don’t know where to get the information and how to go about it*. *We need to improve the awareness*, *especially to the elderly*.*’*–Community Volunteer *‘You all should do something for people to know more*, *maybe a seminar*. *Aunties do not have much information; we are not educated and do not know BC’s seriousness*.*’*–FGD Indian
	Support groups	*‘As time has gone by*, *we did have caring and sharing*, *but it was different*. *Caring and Sharing is done in other ways*. *Sewing*, *whereby they sit down together and talk*, *as they sew they will talk*.*’*–BCSS member *‘Mostly they gave sharing and caring*, *then they gave awareness*, *they helped so much with my family*, *giving moral support*.*’*–BCSS member *‘The BCSS gave sharing and caring support*, *they gave awareness and moral support*.*’*–Community Volunteer
	School awareness programmes	*‘Secondary school would be the best*, *because since secondary school they are exposed to science subjects*, *I think it is better*.*’*–Government hospital doctor *‘We do all talks and awareness campaign whereby we teach them how to do BSE*. *It is very important for finding the initial stage*, *early detection*. *In this way we would teach them the way*. *So we would be organising awareness campaigns in schools or in anywhere*.*’*–BCSS member
	Address stigma and embarrassment about breast cancer	*‘I think less for breast cancer or even cervical cancer*, *it’s like the talk they make it very very open*, *like public*, *so everybody wouldn’t feel shy anymore you know*, *like breasts*, *don’t know*, *feel shy and embarassed to go and ask*, *but once we open*, *public*, *then they will not feel any shy*, *they can ask anybody*, *they can discuss among themselves and also openly*.*’*–Private clinic doctor
	Target husbands to support women in seeking breast health-care	*‘It would be a wise action [to involve men in how to offer breast cancer support]*. *But they don’t know whether the men would attend or not*. *At least they would be more aware*.*’* FGD Chinese *‘…they must ask permission from their husband*. *The husband says ‘no need to go’*, *sometimes it is like that*. *Because men sometimes don’t have good knowledge*.*’* Community Volunteer
3.2 Links and networks between public healthcare personnel and community	Improve currently sporadic BSE education at primary and secondary care level	*‘We always have talks and bring the pamphlets and show everybody*, *and sometimes we ask those who did mammogram to spread the word with their neighbours*.*’*–LPPKN nurse *‘Sometimes the BCSS invites us to give talks*. *Other than that*, *we also collaborate with political parties such as UMNO*, *and with the club for wives of policemen*.*‘*–Government hospital doctor *‘When patient landed with us*, *straight away we teach them breast self-examination*, *they have to do it in front of mirror*.’–Government hospital doctor *‘Yeah*, *I will demonstrate and teach them breast self-examination to the female patients who are around the age where they should be practicing that*.*’*–Private clinic doctor
	Provide financial support, i.e. subsidised mammograms	*‘We must increase our budget to perform mammogram because it’s always high demand*, *so if we have the budget*, *we should increase the subsidy quota for mammogram screening*.*’*–LPPKN nurse

BCare–Breast Cancer unit in Hospital Segamat; MOH–Ministry of Health, IIUM–International Islamic University of Malaya (IIUM has a Specialised Breast Centre)

### 1 Challenges

#### 1.1 Primary care limitations (in terms of the extent to which it can provide gender sensitive and targeted CBE screening services)

The primary health care service (i.e. general practice clinics and family planning clinics in Malaysia) is the first call of contact for patients with acute or chronic illnesses as well as for preventative medicine. Its role in BC early detection is to facilitate biannual CBEs to eligible women and to teach women BSE. A major challenge reported by nurses and doctors to facilitating CBE and BSE was the high workload of staff and, and hence, a lack of time to discuss preventative screening with asymptomatic patients. Some doctors also commented on the variable skills of nurses to detect abnormalities during a CBE, particularly amongst junior staff. Long waiting times in clinics and the limited number of female staff for CBE screening in clinics were challenges reported by FGD participants since women reported to feel uncomfortable to have their breasts checked by male staff.

Nurses described that BSE was often taught to women of child-bearing age (aged ≤40 years) during antenatal or postnatal check-ups and mother-child visits. BSE is commonly taught by showing women pictures of the technique without any physical contact due to the conservative nature of the country and the lack of private facilities during community outreach programmes.

#### 1.2 Challenges in providing mammograms in public sector secondary care hospitals

This sub-theme relates to challenges experienced by hospital staff regarding mammogram screening. Similarly to staffing challenges in the primary care sector, there was a lack of staff specialised in BC care, particularly female radiographers that are mandatory for providing mammogram services. Interviewees reported that only about 30% of hospital radiographers were women. Furthermore, the subsidised District Hospital could only offer a limited number of mammograms each month and the number of mammograms has been reduced over the years. This leaves many women, who don’t want to pay or are not able to pay for a mammogram, waiting. Due to budget constraints, the mammogram equipment and other facilities such as air-conditioners necessary for its operation were old or not always well maintained and could breakdown easily. FGD participants and nurses also reported that women often lacked knowledge about the importance of mammograms and the health care staff in the public sector did not have the time to provide detailed explanations due to a high patient volume and work load.

#### 1.3 A disconnection between primary care-oriented BC screening pathway and secondary care

Hospital physicians described that community clinics had to meet monthly appointment/ screening key performance indicators (KPIs) for CBE that were set by the government. Therefore, BC screening services offered to patients directly from the hospital were not supported by the primary care providers, thus reducing referrals and extending waiting times for patients.

### 2 Barriers

#### 2.1 Personal experiences and barriers to help-seeking

Personal barriers were most discussed by women participating in the FGDs and also recognised by a number of health care professionals. Participants reported that many women from rural areas in Malaysia have very limited knowledge and awareness about BC, did not want to know about it (denial), were misinformed about cancer or cancer treatment or did not think that they were susceptible to getting BC and therefore were not interested in screening. Some women believed in treating cancer with traditional medicine, either alone or in combination with Western medicine, particularly women from the Orang Asli community. Other ethnic differences in terms of breast health seeking were also reported—mainly that Malay women must see a female nurse or doctor for BC screening, whilst Indian and Chinese women can see male doctors. Chinese Malaysian women were also reported by health care staff to be most interested in preventative health testing compared to other Malaysian women.

Malay women reported that some husbands did not permit their wives to show their body to another man and feared that their husband may leave them after a breast removal surgery. Women from all ethnicities expressed embarrassment to show their body to a male doctor or to talk about breast health issues. Child care was a competing priority for some women who said they would not have time for breast checks. FGD and interview participants described that some women were scared of being diagnosed with cancer or that the screening was painful. General practitioners and BC survivors also reported that patients are afraid of breast removal surgery and of losing hair due to BC treatment.

Community volunteers described that some rural women are illiterate and therefore not able to engage with written information (e.g. brochures etc). Language barriers were an issue if health care provider did not speak the primary language of the patients.

Community access barriers, such as having to pay for mammograms was another barrier to participate in screening, as well as difficulties in travelling to the clinic or hospital for CBEs/mammograms and multiple visits needed were reported by nurses, volunteers, doctors and cancer survivors.

### 3 Opportunities

#### 3.1 Community civil society activities

Non-Governmental Organisations (NGOs), community volunteers and nurses from the Ministry of Women, Family and Community Development ran community outreach events and awareness campaigns to educate on BC and demonstrate BSE in the past, however, participants felt that more could be done to increase awareness, particular in rural areas and amongst the elderly. Clinicians suggested that the stigma around BC needs to be addressed so that women feel comfortable talking openly about their breast health. FGD participants and community volunteers advocated for male relatives to be included in BC screening and early detection campaigns, in particular husbands who often act as the decision maker for their wives. NGO representatives and physicians recommended breast health education to be taught in secondary schools for girls to be educated early. NGO programmes that provided moral support for women were thought of as very beneficial to support women who are worried about BC as well as BC patients.

#### 3.2 Links and networks between public healthcare personnel and community

Hospital and clinic staff reported teaching BSE to women who visited government clinics and hospitals, and nurses described health awareness talks that took place in the clinics regularly. Most clinics provide educational brochures with information on BC as part of the awareness talks. Clinicians also and community volunteer collaborated with political parties to receive additional support financial or logistical support (e.g. venue hire). Nurses strongly advocated for an increased number of subsidised mammograms as an incentive to participate in screening.

Overall, awareness raising efforts were depending on the individual clinics, health care staff and clinic budgets and it was suggested to improve the sporadic BSE education within primary and secondary health care settings.

## Discussion

The main finding of our study highlights the challenges faced by health systems and community members in sustaining BC early detection. This is one of the few studies that explored the context of BC screening and early diagnosis in a semi-rural area in Malaysia.

Themes spanned across different levels of the health care system and a number of individual barriers were also identified. A major challenge that was reflected across the themes was that screening was opportunistic and partly targeted at age groups not at-risk of developing BC, and therefore women were not routinely educated and prompted to participate in screening and screening services were not adequately resourced to accommodate large numbers of CBEs and mammograms. This was previously recognised as a barrier to cervical screening by healthcare providers practicing in urban areas in Malaysia [21]. Some individual-level barriers also overlapped with those associated with cervical screening, i.e. lack of awareness, low perceived risk, embarrassment, fear of a cancer diagnosis, competing responsibilities and lack of family support [21]. Fear of a cancer diagnosis was also the most commonly reported barrier to screening in Malaysian women previously (75%) [14]. This suggests that women in Malaysia, regardless of location, face similar barriers to access female cancer screening.

A challenge amongst women from rural areas (including women who were interviewed for this study), where general health literacy is commonly lower, particularly in developing countries [22], is the use of traditional medicine [23], especially amongst the Aborigine community. The use of complementary and alternative medicine has previously been linked to a delay in BC diagnosis and treatment [24]. Previous research also highlighted racial discrimination in hospitals (i.e. non-Orang Asli staff were employed in a government-run Orang Asli hospital), which may have led to distrusting Western Medicine amongst the Orang Asli [25] and may be a major barrier to help-seeking for BC screening. It is therefore important to recognise and address women’s preference for medical treatment and find a compromise in working with traditional and Western medicine practitioners. Lower health literacy amongst rural women may also explain low BC awareness and low perceived susceptibility to getting BC in this study population [26]. Similarly, longer travel distances to a health clinic was a common access barrier amongst rural population groups as reported in this study. Higher education and shorter travel distances to health services were previously linked to better awareness and screening uptake amongst women in Indonesia [27]. Perceived difficulties to access medical support was previously associated with an anticipated delay to seeking medical help for cancer symptoms of over 2 weeks in Malaysia [28].

Due to the ethnic profile of the Malaysian population, health care providers face unique challenges. Findings from the FGDs suggested that the religious, linguistic and ethnic differences affect women’s health seeking needs. Written information is not always provided in all three main languages and Chinese and Indian women who are not fluent in Malay may be disadvantaged when seeking medical help from health care facilities with prominently Malay speaking health care providers. Despite that, Chinese Malaysians are known for engaging better in preventative health care measures compared to other ethnic groups. Malay women in this study reported cultural barriers to BC screening related to the conservative culture. The need to seek permission from their husbands to expose their body to a doctor and the fear of their husbands leaving them if their breasts had to be surgically removed was a barrier for mainly Malay women. Muslim men from conservative cultures often influence women’s decisions and behaviours, which is a major sociocultural obstacle to BC screening and good knowledge about BC amongst husbands was previously associated to their wife’s mammography screening history in Saudi Arabia [29]. This strongly suggests that men also need to be targeted in BC education and screening programmes to improve uptake amongst Muslim communities. Furthermore, training lay health workers form the local community in breast health care may be a low-cost solution to support the varied cultural needs of women in Malaysia and support women to attend BC screening. As such, community health workers have filled in roles to educate women and navigate them to BC screening and conduct CBEs [30].

Women from all ethnic groups reported that they were more comfortable with female health care staff conducting the BC screening and health care providers reported a lack of particularly female support staff to conduct screening (i.e. nurses and radiographers). This was similar to quantitative findings reported previously where 50% women from Segamat reported that male doctors pose a barrier to BC screening attendance [14]. Since BC screening has to be conducted by female radiographers in Malaysia, this likely leads to underuse of mammogram facilities. The Malaysian government should train more female radiographers and nurses specialised in BC screening and early detection. There was also a discrepancy between the need for demonstrating BSE and conducting CBE and the lack of privacy during community screening programmes. Furthermore, women’s financial concerns also opposed the reduction in subsidised mammogram available each month. This is in line with previous research suggesting 70% of women see cost as a barrier to screening [14]. These issues highlight the conflicting reality between women’s personal barriers that are exacerbated by the shortcomings in the healthcare system that have been highlighted in this study [31].

Efforts have been made in the past to increase BC education and screening in rural Malaysia by NGOs, commercial businesses and researchers but awareness campaigns were usually of short duration, and hence, not sustainable [32]. A 5-week mass media campaign has previously been demonstrated to increase BC awareness in urban and semi-urban areas in Malaysia but it seemed to have no impact on BC screening [33]. This study suggested that one-to-one education about BSE was mostly taught to women of childbearing age, which is not the age group most at risk for BC. More sustainable solutions need to be identified and implemented to improve education, BC screening and diagnosis for women who are at average risk for BC.

The rich data collected from a range of different health professionals and women from different ethnic backgrounds in Malaysia is a strength of this study. A limitation of this study is that and women who agreed to participate in this study maybe be more health conscious than other women. However, the sampling of women from the community rather than a health care setting in our study is likely to provide a more representative sample [23].

## Conclusion

Findings from this study highlight a number of health-system and personal barriers that help explain the low BC screening rate amongst Malaysian women. Access to BC screening and early detection services is a major concern for women in semi-rural Malaysia, due to travel, financial and socio-cultural barriers. Opportunities lie in aligning priorities of the different levels of healthcare services and prioritising access to screening through offering culturally appropriate support for women to address embarrassment, fear and BC awareness amongst women and spouses. Opportunities to provide low-cost screening e.g. CBE or other evidence-based screening methods are needed to improve access for semi-rural women in Malaysia. This study is part of a collaborative programme of research about the early detection of BC in Malaysia that comprises several studies including reviews, surveys and the evaluation of efforts to improve the uptake of BC screening. The results of this qualitative study will be triangulated with the results from other studies in the programme including the quantitative analysis of survey data incorporating validated scales.
